# NANETS Guidelines for the diagnosis and management of stage I–III rectal neuroendocrine tumors

**DOI:** 10.1530/ERC-25-0303

**Published:** 2026-02-26

**Authors:** Hagen Kennecke, Ebrahim Delpassand, Seth Felder, Ferga Gleeson, Julie Hallet, David Horowitz, Martin Hyrcza, Bryson W Katona, Maria Kiely, Michelle Kim, Nadine Mallak, Vicky Parkins, Madhulika G Varma, Janice Pasieka

**Affiliations:** ^1^ Division of Medical Oncology and Hematology, Knight Cancer Institute, Oregon Health and Science University, Portland, Oregon, USA; ^2^ Department of Nuclear Medicine, Excel Diagnostics & Nuclear Oncology Center, Houston, Texas, USA; ^3^ Department of Gastrointestinal Surgical Oncology, H. Lee Moffitt Cancer Center and Research Institute, Tampa, Florida, USA; ^4^ Department of Gastroenterology, Mayo Clinic, Rochester, Minnesota, USA; ^5^ Department of Surgery, Sunnybrook Research Institute, Toronto, Ontario, Canada; ^6^ Department of Radiation Oncology, Columbia University Irving Medical Center, New York, New York, USA; ^7^ Department of Pathology and Laboratory Medicine, Arnie Charbonneau Cancer Institute, University of Calgary, Calgary, Alberta, Canada; ^8^ Department of Gastroenterology and Hepatology, University of Pennsylvania School of Medicine, Philadelphia, Pennsylvania, USA; ^9^ Colon and Rectal Surgery, Johns Hopkins Medicine, Baltimore, Maryland, USA; ^10^ Department of Gastroenterology, Cleveland Clinic, Cleveland, Ohio, USA; ^11^ Department of Diagnostic Radiology, Oregon Health and Science University, Portland, Oregon, USA; ^12^ Department of Endocrinology, University of Calgary, Calgary, Alberta, Canada; ^13^ Department of Surgery, University of California San Francisco, San Francisco, California, USA; ^14^ Department of Surgical Oncology, University of Calgary, Calgary, Alberta, Canada

**Keywords:** rectal NET, neuroendocrine, rectal, guidelines, staging, follow-up, NANETS

## Abstract

Well-differentiated rectal neuroendocrine tumors (rNETs) are among the most common NETs and account for approximately 12–27% of all gastrointestinal NETs in North America. Significant discrepancies persist in the management of NETs regarding surveillance strategies, staging modalities, high-risk features, and criteria for surgical intervention. This guideline updates current practices of stage I–III rectal NETs with the utilization of GRADE (Grading of Recommendations, Assessment, Development, and Evaluation) methodology and Delphi method of consensus among leading experts in the North American region. We found that several technological advances, such as ^68^Ga- or ^64^Cu-DOTATATE SSTR PET/CT, and broad adoption of pelvic MRI have improved staging of rNETs, along with modified endoscopic mucosal and submucosal resection and full-thickness excision techniques that demonstrate efficacy and safety for resection. Pivotal long-term outcome studies provide insight into i) risk factors for regional lymph node metastasis, ii) the impact of R1 excision (endoscopic), iii) best practices for intermediate-sized rNETs (11–20 mm), and iv) risk in small rNETs (≤10 mm). Recommendations were developed upon evidence-based conclusions from the GRADE review to define the role of baseline staging with MRI, advanced endoscopy, and transanal endoscopic surgical methods appropriate for T1 rNETs, the role of salvage therapy in cases of R1 resection, and the consideration of pathologic variables to direct definitive treatment and surveillance. We conclude that advances in screening programs and imaging allow for improved detection and staging of rNETs, while long-term outcome studies can better direct patients toward evidence-based treatment management and rectal organ preservation through less radical resection methods.

## Introduction

Rectal neuroendocrine tumors (rNETs) are rare neoplasms that arise from the neuroendocrine cells of the rectal mucosa, accounting for approximately 1–2% of all rectal malignancies. Despite their relatively low incidence, the detection of rNETs has been increasing due to the widespread use of colorectal cancer screening programs and advances in endoscopic technology (1). The majority of rNETs are well-differentiated and slow-growing; however, a subset of these tumors, between 10 and 20%, may exhibit aggressive behavior, with a higher likelihood of regional mesorectal lymph node (LN) involvement and distant metastasis.

Staging of rectal NETs is critical for guiding treatment decisions and predicting long-term outcomes. The American Joint Committee on Cancer (AJCC) and World Health Organization (WHO) staging systems are commonly used to classify rectal NETs based on tumor size, depth of invasion, lymph node involvement, and distant metastasis. In early-stage disease (stages I–III), the primary goals of treatment are complete tumor resection, preserving rectal function and minimizing the risk of recurrence. The management strategy currently depends on the size, grade, and status of lymphovascular invasion within the tumor. Endoscopic resection is typically considered suitable for small, low-grade tumors, whereas larger or more aggressive tumors may require surgical resection with formal mesorectal excision and lymphadenectomy. Along with transanal local excision (LE) techniques, advanced endoscopic techniques, including modified endoscopic mucosal resection (mEMR), endoscopic submucosal dissection (ESD), and endoscopic full-thickness resection (EFTR), are increasingly applied in the management of rNETs ≤ 2 cm in size to allow organ preservation for the vast majority of patients.

Despite the availability of multiple guidelines from organizations such as the European Neuroendocrine Tumor Society (ENETS) (2) and the National Comprehensive Cancer Network (NCCN) (3), significant discrepancies persist regarding surveillance strategies, staging modalities, high-risk features, and criteria for surgical intervention. Meanwhile, several advances, such as i) the introduction of ^68^Ga- or ^64^Cu-DOTATATE somatostatin receptor-targeted (SSTR) PET/CT in the staging of rNETs, ii) long-term follow-up and outcome studies that are more accurately linked to overall survival to provide an evidence-based refinement of high-risk factors, and iii) rapid advancements in modified EMR, ESD, and EFTR methods with safety and efficacy studies, have necessitated an urgent need for updated, evidence-based guidelines.

These guidelines aim to provide a comprehensive adoption of recent advancements and research in the field that impact the diagnosis, treatment, and surveillance of rectal NETs, optimizing clinical outcomes by tailoring treatment approaches based on tumor size, grade, and nodal status while reducing morbidity and preserving quality of life (QOL) by minimizing unnecessary interventions. To achieve this, this manuscript considers current treatment recommendations and reevaluates them in relation to new evidence in accordance with GRADE (Grading of Recommendations, Assessment, Development, and Evaluation) methodology and expert opinion garnered through a Delphi method of consensus among leading practitioners in the North American region.

## Methods

This guideline was developed in accordance with published recommendations for improving the reporting of clinical practice guidelines (4) and follows principles endorsed by the EQUATOR Network (https://www.equator-network.org/). The development process included i) reviewing and summarizing current practices, ii) conducting systematic literature searches to identify updated evidence, iii) developing and reviewing evidence tables, and iv) formulating recommendations through expert consensus using a Delphi process.

Guideline panel members were nominated by the NANETS Guidelines Committee and approved by the NANETS Board based on their recognized expertise in rectal neuroendocrine tumors and disciplines. The selection process also emphasized diversity, equity, and minimization of potential conflicts of interest. External peer review and patient stakeholder input were sought to ensure the recommendations are scientifically valid and aligned with patient priorities. External and patient reviewers are listed in the ‘Acknowledgments’ section.

**Figure 1:**
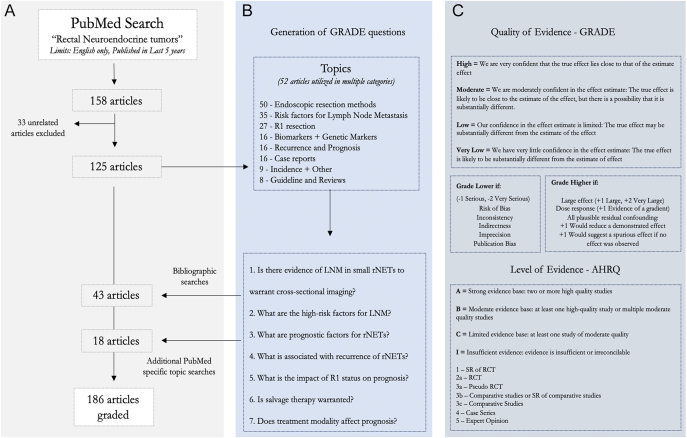
Flowchart and grading process outlined. (A) PubMed search and graded article origin, (B) generation of GRADE questions, and (C) evidence and recommendation grading. Original PubMed search was used to derive recent evidence and to derive advancements in the field. Generation of GRADE questions allowed for further PubMed searches centered on the specific topics to retrieve relevant historical research literature/studies. Articles were graded based upon the type of articles and strength (1, 2, 3, 4, 5), conclusion statements were graded, and relevant conclusions and grades were considered with Delphi consensus statements to derive the cumulative strength of the recommendations as considered in GRADE values of high–very low designating degree of certainty of recommendation. Abbreviations: GRADE, Grading of Recommendations Assessment, Development, and Evaluation; LNM, lymph node metastasis; rNETs, rectal neuroendocrine tumors; and R1, positive resection margins.

Current practices for rectal NET management were reviewed and summarized from multiple existing guidelines, including NANETS (5), ENETS (2), NCCN (3), and AJCC (6). An extensive bibliographical search was conducted to develop evidence tables and summarize recommendations. To update the evidence tables, a PubMed search was conducted; the PubMed bibliographical search was updated as of January 2025 using the term ‘rectal neuroendocrine tumors’ with the limit of a published date of ‘within 5 years’ and availability in the English language as summarized in Fig. 1A. Articles were reviewed for relevance to current treatment paradigms and advances in the field. Each article was reviewed and graded in accordance with the GRADE (Grading of Recommendations, Assessment, Development, and Evaluation); levels of evidence were concluded after a review (7) with grading standards listed in Fig. 1B and C and then incorporated into evidence tables with summaries of relevant findings, conclusions, limitations, and bias.

**Figure 2:**
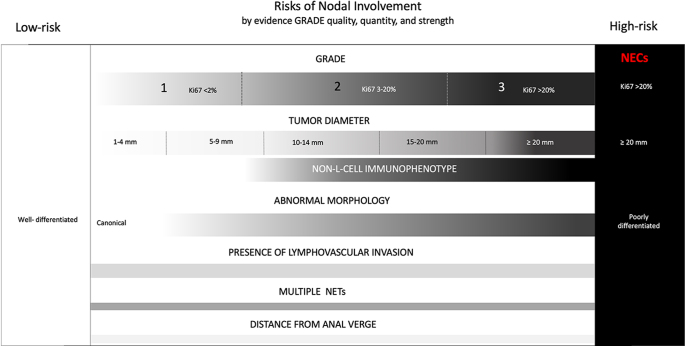
Risk factors for rectal NETs. Below is a representation of the evidence reviewed in the development of this guideline for the risk of lymph node involvement. Evidence suggests that the prognosis of rectal NETs in broad strokes lies on a spectrum with small, well-differentiated tumors on one end with 5-year OS estimated at approximately 99% and large, grade 3 NETs with 5-year OS estimated at 35%. As parameters such as tumor size, grade, tumor extent, presence of lymphovascular invasion and characteristics such as abnormal tumor morphology increase, so does risk for nodal involvement as they represent progression along the spectrum. In multivariate analysis, tumors size and grade remain independent risk indicators of LNM and non-L-cell phenotype in tumors larger than 10 mm. Abbreviations: mm, millimeter; NET, neuroendocrine tumor; NEC, neuroendocrine carcinoma.

A Delphi approach (8) was chosen to achieve group consensus by conducting iterative rounds of questionnaires and allowing group discussion. The stated objective was to identify consensus in areas relevant to the diagnosis and management of non-metastatic rectal NETs. A total of 14 Delphi members representing medical oncology (1), nuclear medicine (2), surgery (5), gastroenterology (3), pathology (1), endocrinology (1), and radiation oncology (1) were included. Delphi structure and terms were established in the initial meeting, and a total of six meetings were held. Delphi members were divided into five subject matter working groups to review the key literature and current guidelines. The groups identified 62 statements to be surveyed in the areas of diagnosis, clinical and pathologic risk features, imaging and staging, endoscopic and surgical management, adjuvant therapy, and surveillance. Survey statements were sent out electronically after a virtual meeting on April 26, 2022, and results were discussed at the in-person NANETS annual meeting held on October 27–29, 2022, in Washington, DC. Selected questions were edited for clarification, and all 62 statements were sent out electronically after the in-person meeting. Results of the second survey were discussed at a virtual meeting held on July 10, 2023. In a group meeting on February 23, 2024, Delphi members identified 12 statements, which did not achieve consensus, to be resurveyed in a third and final survey. There were 13/13 (100%) respondents to the first survey, 13/14 (93%) to the second survey, and 11/14 (79%) to the third survey. A scale of 1–9 was chosen, with 1 indicating total disagreement and 9 indicating total agreement. All responses were confidential. Central tendency was measured by the median score, with a score of 7–9 indicating agreement (support), a score of 4–6 indicating a neutral response, and a score of 1–3 indicating disagreement (lack of support). Consensus was predefined as 70% of respondents scoring within any 3-point range.

### Delphi members

H Kennecke (Medical Oncology, Chair), Ebrahim Delpassand (Nuclear Medicine), Seth Felder (Colorectal Surgery), Ferga Gleeson (Gastroenterology), Julie Hallet (Surgical Oncology), David Horowitz (Radiation Oncology), Martin Hyrcza (Anatomic Pathology), Bryson Katona (Gastroenterology), Maria Kiely (Colorectal Surgery), Michelle Kim (Gastroenterology), Nadine Mallak (Nuclear Medicine), Janice Pasieka (Surgical Oncology), Vicky Parkins (Endocrinology), and Madhulika Varma (Colorectal Surgery).

## Results

A total of 158 articles were retrieved from the PubMed search, of which 125 were included. An additional 61 articles were included from the bibliographical search and additional PubMed specific topic searches with MeSH ‘<category name> and rectal neuroendocrine tumors’ from inception to January 2025. Guideline conclusion statements were classified by GRADE evidence, and management recommendations were developed to reflect updates in knowledge agreed upon by Delphi consensus. Figure 1A is a flow chart of the PubMed search of the generation of graded articles.

### Incidence

Our knowledge of rectal NET epidemiology in North America primarily comes from the SEER database (9) and the Ontario Cancer Registry (10). The SEER database estimates an incidence of 1.04 cases per 100,000 (11), and the Ontario Cancer Registry has a similar incidence of 0.74 cases per 100,000 (10, 11). Both sources identify rectal NETs as among the most common NET type, with SEER estimating that they comprise 17.7% of all NETs (12).

The continuous rise in rectal NET incidence (1975–2012) is attributed to improved diagnostic methods, such as endoscopic screening, which have led to greater detection of asymptomatic and smaller lesions (13), rather than an actual increase in cases, as the mean age of diagnosis (54 years) has remained stable (14).

Ethnic variation in incidence is notable, with Asian/Pacific Islanders (41%) being four times more likely to be diagnosed than white patients (12%) in SEER data (1). Similar trends in Taiwan, Korea, Japan, and China suggest a genetic rather than environmental cause, unaffected by migration (15, 16, 17, 18, 19, 20, 21). While incidence is also elevated in African Americans and Hispanics, the absence of data from Africa and South America makes it unclear whether access or socioeconomic factors play a role (1).

#### Conclusion

- Improved diagnostic methods have identified more cases of asymptomatic small lesions *(3b/A)*.
- Higher rates of rectal NETs are seen in individuals of Asian/Pacific Islander ancestry *(3b/A)*.

### Pathology

The pathologist report is guided by the protocol for the examination of specimens from patients with neuroendocrine tumors of the appendix from the College of American Pathologists (22). In addition, rNETs may be classified into two major biological cell types: L-cell and non-L-cell types (predominantly composed of E-cell types) (23, 24, 25, 26, 27, 28). L-cell NETs constitute approximately 80% of rNETs (22, 25, 28, 29). They produce pancreatic polypeptide (PP) and peptide YY (PYY) and are characterized by a trabecular, ribbon-like, or pseudo-glandular architecture (24). EC-cell NETs are serotonin-producing tumors that share morphological characteristics with their small intestinal and colonic counterparts. While these cell types do not always exhibit distinct clinical behavior, evidence suggests a strong association between the E-cell phenotype and more aggressive disease features (23, 24, 25, 26, 27).

To determine L-cell origin, they can be stained for glucagon-like protein (GLP-1) and/or peptide YY (PYY)/PP – although as not all pathology laboratories have these stains, L-cell origin can be convincingly inferred by CDX2 and serotonin negativity (24).

### Clinical presentation

Rectal NETs typically appear as small (≤10 mm), smooth, round, mobile submucosal nodules with a yellowish mucosal covering due to the presence of chromogranin. They commonly arise 4–8 cm from the anal verge from the rectal mucosa, and some retrospective studies suggest that a distance greater than 7 cm may be an independent risk factor for LNM (30). While this is the standard presentation, some lesions exhibit ulceration, erosion, umbilication, hyperemia, or semi-pedunculation, which may correlate with aggressive disease features (31, 32, 33).

Most rectal NETs are small, with up to 90% of lesions measuring <1 cm in diameter (6). They are typically diagnosed incidentally through colorectal cancer screening with an incidence of approximately 1 in every 1,500 colonoscopies (34). Approximately 50% are removed during the index endoscopy, as they are often mistaken for hyperplastic polyps and are only diagnosed as rectal NETs through histopathological analysis (35). In fact, one study reported that only 18% of experienced endoscopists suspected rectal NETs visually during endoscopic examination, highlighting a persistent diagnostic challenge (36). This results in two distinct diagnostic and treatment pathways for patients with rNETs, and many patients may be initially treated with incomplete excision at index presentation prior to full staging.

Therefore, in clinical practice, diagnosis typically follows two pathways:

- Suspected rectal NETs – identified during colonoscopy, leading to targeted biopsy.
- Unrecognized at initial resection – initially removed as presumed hyperplastic polyps or adenomas and later confirmed as rNETs via histopathological analysis.

#### Conclusion

- Rectal NETs often present endoscopically as small, yellowish, submucosal nodules but can have varied morphological features *(3b/A)*.
- Misdiagnosis of rectal NETs at the initial endoscopy is common and is expected to remain a challenge, necessitating its integration into the overall management approach for rectal NETs *(3b/A)*.

### Staging

The World Health Organization (2019) assesses the risk of metastasis of rectal NETs by the size of the tumor, the depth of invasion, increased mitotic index, and lymphovascular invasion. As detailed in Table 1A, the grading of rectal NETs is based on mitotic count per 10 high-power fields (HPFs) and the expression of Ki-67, a tumor proliferation marker, in a hot spot composed of a minimum of 500 cells and ideally 1,000–2,000 cells.

**Table 1:** Grading and TNM staging for rectal NETs. Based upon WHO (2019) grading and AJCC 9 (2024) staging recommendations for rectal NETs.

**A. Grading for rectal NETs (WHO, 2019)**			
	Low grade	Intermediate grade	High grade
Grading	G1	G2	G3
Mitotic count (mitoses/10 HPFs)	<2	2–20	>20
	**AND**	**OR**	**OR**
Ki-67 index	<3%	3–20%	>20%

**B. TNM staging (AJCC 9, 2024)**			
T stage	T description	N/M stage	N and M description
TX	Primary tumor cannot be assessed	Nx	Lymph node status not detected
T0	No evidence of primary tumor metastasis	N0	Absence of lymph node
T1	Tumor ≤ 2 cm, invades lamina propria or submucosa	N1	Regional lymph node metastasis
T1a	Tumor ≤ 1 cm	Mx	Distant metastasis not detected
T1b	Tumor > 1–2 cm	M0	No distant metastasis
T2	Tumor > 2 cm or that invades muscularis propria	M1a	Distant metastasis – liver only
T3	Subserosa or perirectal tissues	M1b	Distant metastasis – extra-hepatic only
T4	Serosa or other organs	M1c	Distant metastasis – liver and extra-hepatic

Abbreviations: AJCC, American Joint Committee on Cancer; HPFs, high power fields; N, node; NETs, neuroendocrine tumors; M, metastasis; T, tumor; TNM, tumor node metastasis; and WHO, World Health Organization.

In the AJCC version 9 (as detailed in Table 1B), the clinical and pathological stage classifications are determined based on tumor size and/or extent (T), the presence of regional lymph node metastasis (N), and the presence of distant metastasis (M). For rNETs, it is recognized that many lesions will be managed endoscopically; thus, T1NX is recognized as stage 1. The latest AJCC version 9 staging protocols offer detailed guidelines for staging of GEP-NET patients (3).

#### Imaging

Imaging plays a crucial role in the staging of rectal neuroendocrine tumors (NETs) by assessing tumor size, depth of invasion, regional/mesorectal lymph node involvement, and distant metastases.

There is wide evidence and strong support to suggest that baseline imaging with MRI will provide more complete staging that EUS does not permit. MRI offers superior soft tissue contrast and is highly sensitive in detecting mesorectal and distant lymph node involvement, which is critical for accurate staging. Unlike EUS, MRI provides whole-pelvis evaluation, allowing for better assessment of potential metastatic spread beyond the rectal wall, which is crucial for treatment planning. Most evidence derives from low-grade evidence of expert opinion (6, 37, 38) and retrospective studies (39, 40, 41, 42, 43). The use of MRI is bolstered by a prospective study that found 25% of patients with small (<10 mm) rectal NETs were found to have LNM on pelvic MRI and confirmed by ^68^Ga-DOTATATE somatostatin receptor-targeted (SSTR) PET/CT (44). Evidence indicates that small (<10 mm) rNETs should not be presumed as indolent (39, 40, 41, 42, 45, 46) and warrant complete baseline staging with MRI to rule out LNM.

In the case of MRI abnormalities, the use of ^68^Ga- or ^64^Cu-DOTATATE (SSTR) PET/CT to rule out or identify lymph node (LN) or distant metastasis is strongly supported by the evidence and has revolutionized staging by identifying tumors with high somatostatin receptor expression, such as in the case of well-differentiated rNETs (47). Somatostatin receptor expression is a key biomarker for rectal NETs (rNETs), as most well-differentiated rNETs overexpress somatostatin receptors (particularly SSTR2) (27, 48, 49). This makes SSTR-based imaging highly sensitive for both staging and therapy selection. SSTR-PET/CT imaging offers superior sensitivity and specificity for detecting metastatic or recurrent disease compared to conventional imaging modalities, particularly for gastroenteropancreatic NETs (GEP-NETs) (50, 51). Whether SSTR-PET/CT imaging could be used instead of conventional MRI-DWI and CT as a sole regional staging modality has not yet been defined in clinical practice. Evidence supports that this modality is particularly useful for staging well-differentiated NETs and for selecting candidates for peptide receptor radionuclide therapy (PRRT) (14, 37, 38, 47, 52, 53, 54, 55, 56, 57, 58, 59, 60, 61). PRRT eligibility depends on several factors, including receptor expression, tumor burden, and overall disease status (52). High uptake on SSTR-PET/CT is a strong predictor of response to PRRT, and combining it with FDG-PET imaging can provide valuable information about tumor aggressiveness, particularly in higher-grade lesions (62).

For patients with T2 tumors, multiphase contrast CT scan of the chest, abdomen, and pelvic (CAP) is an essential component of the staging workup for assessing distant metastases and structural disease involvement (47). For well-differentiated rNETs, high somatostatin receptor expression correlates with less aggressive behavior and better prognosis (48). In contrast, large rectal NETs that could be grade 3 should undergo FDG-PET imaging in addition to SSTR-PET/CT, as FDG avidity is more common in higher-grade, poorly differentiated lesions and is associated with a worse prognosis (63, 64). Dual-modality PET/CT imaging (SSTR and FDG) provides complementary information, with FDG uptake indicative of aggressive biology and SSTR uptake guiding targeted therapy decisions (64).

#### Conclusion

- Pelvic DW-MRI is recommended for staging to identify indications of LNM in tumors 5 mm or greater *(5/A)*.
- ^68^Ga- or ^64^Cu-DOTATATE SSTR PET/CT is effectively used to confirm the presence of LNM following abnormal findings on DW-MRI *(3b/A)*.
- The integration of pelvic DW-MRI, ^68^Ga- or ^64^Cu-DOTATATE PET/CT, and abdominopelvic and chest computed tomography (CT) multiphase scans (for T2 tumors) provides a comprehensive staging approach, allowing for precise tumor characterization and more tailored treatment strategies *(4/A)*.

### Prognosis

Long-term outcomes of rectal neuroendocrine tumors (rNETs) are better than NETs at other sites, with an overall 5-year survival rate of approximately 88% for all sizes (65). This favorable prognosis is primarily driven by the predominance of early-stage and low-grade rNETs (66, 67). However, 10–20% of cases will metastasize to regional lymph nodes and beyond (24). Overall survival for rNETs is strongly associated with the degree of nodal involvement, the presence of distant metastasis at diagnosis, and patient age (28, 68, 69).

### Lymph node and distant metastasis

Overall survival is significantly affected by the presence or progression of lymph node and distant metastases. While patients with T1N0M0 and T1NXM0 (stage I) disease have a 5-year OS rate of 98–100%, the presence of regional lymph node metastases (N1) reduces this rate to between 54 and 74%, and distant metastases decrease it further to between 15 and 37% (70, 71, 72, 73).

Staging focuses on identifying the small subset of patients with more aggressive disease that advances from local to regional stages. Greater nodal involvement is an independent prognostic factor for worse outcomes, with a 5-year OS rate of 81.8% for N0 (no nodal involvement) compared to 57.8% for N1 (1–4 nodes) and 31% for N2 (5+ nodes) (74).

Tumor grade, size, extent, and non-L-cell immunophenotype in rNETs > 10 mm have been demonstrated through multivariate analysis to be independent indicators of higher risk of LNM (Fig. 2).

#### Grade

Higher-grade rNETs (G2 and G3) are consistently identified as independent predictors of lymph node metastasis and poorer overall survival. While no single parameter can predict LNM, tumor grade is one of the strongest independent predictors of higher LNM risk (71, 75, 76, 77, 78, 79, 80, 81, 82, 83), distant metastasis (78, 79, 84), and overall survival (71, 79, 84) in multivariate analysis. The 5-year survival rates for patients with G1, G2, and G3 tumors were 87.7, 47.6, and 33.3%, respectively (71).

Research suggests that G2 tumors are more aggressive than G1 tumors, often larger, more invasive, and associated with higher rates of lymphovascular invasion (LVI), perineural invasion (PNI), and positive resection margins (77, 78, 79). Grade consistently is shown to be strongly associated with other clinical features such as LVI, tumor size, and extent that appear significant in univariate analysis, with grade being identified as the only independent predictor in multivariate analysis. Grade has been shown to be particularly effective in stratifying risk of tumors between 10 and 20 mm (76, 78).

#### Tumor size

Tumor size is a well-established independent factor affecting prognosis. The risk of death in patients with tumors >20 mm is more than 10 times higher than in patients with tumors <20 mm (85). While tumor diameter is a strong independent indicator for worse prognosis, overall survival and the risk of LNM metastasis (68, 85, 86, 87) by size are driven ostensibly by tumors over 20 mm (71). There is general consensus that the rate of LN metastasis increases as tumor size increases, with rates of LNM in <10 mm ranging from 3 to 25%, 10–20 mm from 15 to 50%, and >20 mm from 60 to 80% (1, 70). The 5-year/10-year OS rates for tumors stratified by size demonstrating this demarcating split in tumor behavior of <20 mm and over 20 mm can be seen for tumors stratified by size with 1–4 mm 99.6%/99.6%, 5–9 mm 99.4%/99.4%, 10–14 mm 100.0%/97.2%, 15–20 mm 90.6%/90.6%, and over 20 mm 47.3%/35.5% (23). Although the prognosis for tumors <20 mm is generally excellent, not all small tumors (<10 mm) should be presumed low risk (38, 39, 40, 41, 43, 45), and lesions between 11 and 20 mm have a higher rate of LNM than their <10 mm counterparts (88).

#### Non-L-cell immunophenotype

Compared to their counterparts, non-L-cell immunophenotype demonstrates significantly worse survival outcomes, with 5-year and 10-year OS rates of 48.7 and 40.6%, compared to 92.9 and 88.7%, respectively (23). Non-L-cell immunophenotype is linked to higher tumor grade, larger tumor size and extent, LVI, PNI, lymph node (LN) involvement, and distant metastasis (23, 25, 26). While the EC-cell phenotype is strongly associated with worse prognosis, it does not always indicate high risk (23, 25). Multivariate analyses suggest that a combination of non-L-cell phenotype and tumor size greater than 10 mm provides a more reliable predictor of aggressive disease than immunophenotype alone (25).

Although L-cell tumors are generally associated with favorable outcomes (23, 24, 27), they should not be considered benign as a small number of L-cell tumors have developed metastasis, highlighting the need for complete staging and continued follow-up (23).

CgA is typically positive in EC-cell tumors, although due to overlapping expression in L-cell tumors, which can be CgA positive or negative, this is no longer considered a reliable biomarker to direct treatment (24). CgA positivity has been demonstrated in numerous studies to be associated with more aggressive disease and poorer prognosis (27, 69, 89), lending greater evidence to EC-cell or non-L-cell immunophenotypes as high-risk factors in tumors >10 mm.

#### Tumor depth

Depth of invasion also impacts overall survival in rectal NETs, with one study of 849 rectal NETs reporting a 98% 5-year survival rate for tumors confined to the submucosa, compared to 88% for deeper tumors (43). It has been identified in some multivariate analyses as an independent risk factor for LNM (90, 91), although its association with LNM is primarily found in univariate analysis (40, 92) and strongly linked to tumor size and grade (25).

T stage is associated with a 5-year overall survival (OS) of 95.7% for stage 1, 91.1% for stage 2, 85.6% for stage 3, and 49.7% for stage 4 (6). However, since the T stage is dependent on both tumor depth and tumor size, it is not an independent prognostic factor (93).

#### Other associated factors with LNM

Two other notable parameters that have emerged as independent risk factors for LNM include morphological abnormalities (31, 32, 37, 56, 80) and tumor distance from the anal verge >7 cm (78). Of these, morphological abnormalities of the tumor have significant evidence to support them as a risk factor to consider. Tumor distance from the anal verge has only recently been proposed as an independent risk factor for LNM, possibly related to different lymphatic drainage pathways above and below the peritoneal reflection (78); however, this remains inconclusive as this parameter has not been investigated in other studies and these findings have yet to be replicated. In addition, several nomograms have been developed, primarily based on tumor grade, extent, and diameter; however, they have yet to be externally validated (42, 86, 90). The GATIS score was derived using five independent prognostic factors to generate a continuous risk prediction for overall survival (OS) and progression-free survival and has been proposed for use in treatment planning and follow-up intensity of newly diagnosed rectal tumors (94).

### Presence of LVI

Our understanding of the prognostic value of lymphovascular invasion as a risk factor for LNM has changed in the recent years. This is in part due to the increase in the rates of LVI detection secondary to the immunohistochemical methods replacing H&E staining as the primary way pathologists find LVI. A 21-study meta-analysis found that endoscopically treated small (<10 mm) rectal NETs with LVI have an excellent prognosis with only a 0.3% recurrence rate during the 5-year follow-up period (95). With rates of LVI in cohorts of up to 40–50% in small tumors, its clinical significance is questionable, as recurrence and progression remain very low in long-term outcome studies (82, 95). In addition, while there is evidence for LVI as a higher risk parameter for LNM (31, 40, 46, 73, 81, 86, 87, 96), there is equally convincing evidence that it is not (82, 95, 97, 98, 99, 100). LVI presence or absence has been used successfully in combination with other parameters, such as grade, to provide additional context in risk stratification (77).

Figure 1 depicts the varying degree and strength of risk factors for LNM.

#### Conclusion

- More than 90% of rNETs show indolent behavior and can be managed with endoscopic/transanal management *(3b/A)*.
- Tumor grades 2 and 3 are independent risk factors for LNM *(3b/A)*.
- T1a tumors <5 mm are at a lowest risk of relapse, while 5–10 mm tumors have some risk of LNM *(3b/B)*.
- T1b tumors 11–20 mm have higher rates of LNM *(3b/B)*.
- Non-L-cell immunophenotype is a high-risk feature associated with T1b tumors *(3b/B)*.
- Tumors size >20 mm have a significantly worse OS rates *(3b/A)*.

### Recurrence

Long-term outcome studies report low recurrence rates, predominantly ranging from 0 to 4.2% (101, 102, 103), with median recurrence typically occurring 3–5 years after initial diagnosis (45, 85, 92, 94, 101, 102, 104, 105, 106, 107, 108, 109).

Currently, no well-established predictive features of recurrence have been identified by studies evaluating surveillance (82, 104, 106). Only a single study found biopsy resection associated with a higher recurrence rate compared to more advanced endoscopic techniques (27.5%; 11/40 vs 2.8%; 15/527), although it had no impact on overall survival (91). Despite overall low recurrence rates, recurrence has been documented even in low-risk cases (<10 mm, G1, low Ki-67 index) (46, 56), and in long-term outcomes, recurrence is not associated with tumor size, curative resection status (R0 vs R1) (46, 82, 110), LVI (77, 82, 96, 111), or resection method (112, 113, 114, 115, 116); however, there is evidence that suggests grade 2 lesions account for a disproportionate share of recurrence (92). Evidence suggesting that the presence of synchronous NETs leads to higher rates of metachronous NETs is difficult to decipher as it may be confounded with an incomplete index colonoscopy and is not consistently found in long-term studies (40, 104, 106, 117).

In G1 rNETs, lymph node and distant metastases are rare after resection but have been reported (84, 86, 118, 119), although G2 has been suggested to possibly be at more risk (71, 81, 83). In one 10-year follow-up study, all three instances of recurrence involved grade 2 lesions (82), with some support that found in cases of neuroendocrine liver metastasis (NELM) originating from the rectum (rNETs), 71% of cases involved grade 2 lesions (84).

#### Conclusion

- Local recurrence rates for rNETs are generally low (0–5%) and may be up to 10 years or more after initial diagnosis *(3b/A)*.
- Recurrence or progression can occur independently of rNET size *(3b/A)*.
- Grade 2 rNETs account for a disproportionate share of metastatic recurrence *(3b/B)*.
- Biopsy resection only shows higher recurrence rates than patients treated with endoscopic tumor excision *(3b/C)*.

### Re-excision for positive resection margins

While positive resection margins have been considered a high-risk factor for recurrence and poor outcome, there is conflicting evidence to support this conclusion due to an overall low relapse rate and competing determinants of locoregional relapse (111, 120). Research linking positive resection margins with worse prognosis has been attributed to independent variables, such as tumor size (121) and tumor morphology (39, 116). Studies of salvage surgery have reported 30–40% of positive and indeterminable resection margins accurately assessed to have remnant tumors, implying that >60% have no residual microscopic disease after presumed incomplete excision (82, 122). International guidelines have acknowledged this uncertainty (2), and multiple long-term follow-up studies have reported on outcomes of patients treated with surveillance rather than surgery reporting that a positive microscopic resection margin after successful endoscopic resection is an inconsistent predictor of recurrence and may not routinely justify radical surgery (46, 49, 100, 110, 114, 115, 116, 120, 121, 123, 124, 125, 126, 127, 128, 129, 130, 131, 132). Unlike the experience reported in Asian countries, in the west, the scenario of incidental recognition of rNETs is common, and as a result, traditional conventional polypectomy or snare removal is frequently used, which may not allow adequate depth or margins, leading to higher R1/R2 rates and a need for salvage intervention (1). For incidentally diagnosed rNETs with concern of residual tumor, advanced endoscopic techniques should be employed ideally within the timeframe that the original endoscopic scar is still visible to achieve a complete excision with EMR, ESD, or EFTR. Current practices for patients with residual tumor after biopsy or excision suggest the role of more advanced endoscopic resection or transanal endoscopic surgery when feasible to avoid radical surgery.

#### Conclusion

- The independent prognostic impact of microscopic positive margins after successful endoscopic resection is not uniformly established *(3b/A)*.
- The value of total mesorectal excision (TME) surgery is not established for tumors up to 20 mm excised with positive margins in the absence of evidence of nodal metastasis or high-risk features *(3b/A)*.
- More advanced endoscopic resection or full-thickness excision surgery is recommended for 5–20 mm tumors if initial endoscopic management did not achieve negative tumor margins *(3b/A)*.

### Resection methods

Several resection methods are used for rNETs, ranging from routine biopsy of lesions to TME. The choice of resection method depends on several factors, including the endoscopic and surgical experience of the institution. A summary of the different resection methods is presented in Supplementary Table 1 (see section on Supplementary materials given at the end of the article). Assessing resection efficacy has been guided by the ability to achieve R0 resection through complete removal of the tumor, accounting for its depth of invasion and avoiding complications such as bleeding, perforation, and stricture.

### Resection efficacy

The ability to achieve tumor resection is driven primarily by resection method, size, and endoscopic morphology (133). The reported rates of complete resection can vary significantly depending on the experience of the endoscopist or surgeon and the size of the lesion, leading to differences across studies, geographical region, and clinical practices.

#### Misidentified small rectal neuroendocrine tumors (rNETs) and diagnostic biopsies

Hot and cold snare polypectomy is commonly used in the resection of small rNETs that are interpreted as hyperplastic polyps. Due to the submucosal nature of rNETs, *en bloc* resection rates are estimated to range between 20 and 30% with R0 status between 17 and 30% (36, 134, 135, 136). It is noteworthy that diagnostic biopsy can achieve resection in the smallest of lesions with R0 resection rates reported at 100% for 2–3 mm lesions but declines significantly to 34.3% for 4–5 mm lesions and to 0% for 6–10 mm lesions (53).

Endoscopic mucosal resection (EMR) is effective in achieving resection for tumors less than 10 mm, although some mEMR methods such as EMR with circumferential precutting (EMR-P) and underwater endoscopic mucosal resection (UEMR) (see Supplementary Table 1) are not limited by this size constraint. Conventional EMR (cEMR) can achieve resection rates of 95.6–99.0% and R0 rates of 74.5–84.3% (134, 137), although it can also have histologically complete rates as low as 30%. Modified EMR (mEMR) shows improved outcomes with greater consistency, with *en bloc* rates reaching upward of 98.8–100% and R0 rates of 86.2–97.4% (36, 131, 134, 136).

Advanced endoscopic procedures and transanal surgical excision can achieve complete resection for tumors up to 20 mm. Endoscopic submucosal dissection (ESD) can achieve *en bloc* rates of 97.5–100% and R0 rates of 71.2–100%, although these outcomes may vary (35, 133, 135, 138). Hybrid endoscopic submucosal dissection (hybrid-ESD) utilizes modifications to the ESD method and can achieve complete resection of up to 99.0–99.2% and R0 rates of 88.1–94.1% (134, 139). The over-the-scope clip (OTSC; Ovesco Endoscopy AG, Germany) has been developed. In contrast to common endoscopic clips, the OTSC is able to capture a larger volume of tissue with a higher compression force; thus, it can be used for closure of luminal perforations larger than 10 mm (140). Endoscopic full-thickness resection (EFTR) can achieve en bloc rates of 100% and R0 rates of 77.2–100%, (141, 142), and transanal endoscopic surgery can achieve *en bloc* rates of 92% and R0 rates of 91.5–92.0% (142, 143).

Radical resection (RR) includes TME and low anterior resection, and both achieve *en bloc* and R0 resection rates of 100% (142).

## Comparative efficacy to achieve R0

### Conventional EMR vs modified EMR vs ESD

Multiple meta-analyses of 27, 18, 14, 14, and 4 studies, respectively, demonstrated that mEMR and ESD had higher complete resection rates than conventional(c) EMR (135, 144, 145, 146). mEMR achieved complete resection rates comparable to ESD for lesions <10 mm, with significantly shorter procedure times and no increase in adverse events (102, 135, 144, 145, 147). Lesions up to 16 mm showed similar resection rates between EMR and ESD (102), while for lesions between 10 and 20 mm, there were significantly more positive resection margins with EMR, suggesting that ESD should be preferred for lesions in this size range due to the need for salvage therapy (147). ESD was associated with longer procedure times and a higher likelihood of bleeding than EMR (147). There were no differences in recurrence rates across any of the treatment modalities in all meta-analyses (101, 136, 144, 145, 146, 147).

Meta-analyses concluded that EMR, particularly EMR with a ligation device (EMR-L) and cap-assisted EMR (EMR-C) (see Supplementary Table), is preferred to ESD for treating rectal NETs ≤ 10 mm in terms of procedural simplicity, lower skill requirements for endoscopists, lower medical costs, and availability for day surgery (102, 136, 144, 145, 146, 147). Several additional retrospective and observational studies have supported these findings (114, 148, 149).

### ESD vs TEM

A retrospective analysis of 114 rectal NET patients (<2 cm) (ESD = 55; TEM = 59) showed a significantly higher complete resection rate in the TEM group (91.5 vs 70.9%; *P* = 0.005) with no significant difference in recurrence rates, surgical complications, or hospital stays. ESD was associated with a significantly shorter procedure time (27.5 vs 56 min; *P* < 0.001) (143). Similarly, a meta-analysis of three studies involving 158 patients with large rectal tumors (not exclusively NETs) reported comparable resection rates, adverse events, and recurrence rates between ESD and TEM, with ESD linked to shorter procedure times and hospital stays (139).

### EFTR vs TEM

In one study comparing EFTR and TEM for the treatment of rNETs, the en bloc resection rate, R0 resection rate, tumor size, and specimen size were similar between the two groups. However, there was a significant difference in the mean procedure time, with the TEM group averaging 48.9 min compared to 19.2 min in the EFTR group (142).

#### Effect of treatment modality on outcomes

As studies support the success of various resection techniques to achieve comparable histologic margins, this allows the choice of resection method to include considerations of availability and procedural risks and morbidity, including procedure time, hospital stay, recovery time, and QOL. Modified endoscopic mucosal resection (mEMR) for lesions ≤20 mm provide an effective and minimally invasive approach with favorable procedural and recovery outcomes. Studies evaluating rNETs < 10 mm (104, 136, 145, 146, 147, 148, 149, 150, 151, 152, 153, 154, 155), <15 mm (102, 156, 157, 158, 159), and <20 mm (45, 76, 82, 91, 147, 160) support the use of mEMR techniques. Transanal surgery (TEMS and TAMIS) and hybrid ESD, ESD, or EFTR provide options for patients with larger tumors (>10 or >15 mm) and yield equivalent oncologic outcomes with differences in hospital stays and procedural duration.

RR can achieve R0 margins in localized rNETs but introduces greater risks and postoperative morbidity (30, 74, 93, 161). Data from the National Cancer Database (161) identified a higher mortality rate (HR 2.47) associated with RR in 11,929 rectal NET cases. Similarly, Cai *et al*. (93) analyzed 17,308 localized GEP-NET cases from the SEER registry, including 6,622 patients who underwent extended resection for lymph node harvesting, and found no OS benefit in 11–20 mm tumors. A study to evaluate QOL and rectal function among 272 patients with 11–20 mm rectal NETs showed no differences in oncologic outcomes between LE and RR, but LE demonstrated significantly better QOL and anal function (30). The cumulative result of evidence demonstrating similar survival between patients with T1N0M0 and T1NXM0 tumors has led to the revised definition of endoscopically treated patients with T1NXM0 tumors as stage I tumors in the 2024 AJCC version 9 update. This validates current management practice to treat the majority of T1 patients with endoscopic resection without nodal resection (6).

#### Conclusion

- T1a tumors <5 mm are successfully treated with EMR, while 5–10 mm tumors may be offered modified EMR after staging to rule out LNM *(3b/A)*.
- For T1bN0 tumors, advanced endoscopic techniques (ESD, EFTR, and TES) are prioritized, while modified EMR shows favorable outcomes, with a lower procedure time, hospital stay, and lower cost, and is appropriate for low-risk T1b tumors *(3b/A)*.
- En bloc and R0 resections vary widely on expertise level across studies *(3b/A)*.

## Recommendations

**Figure 3:**
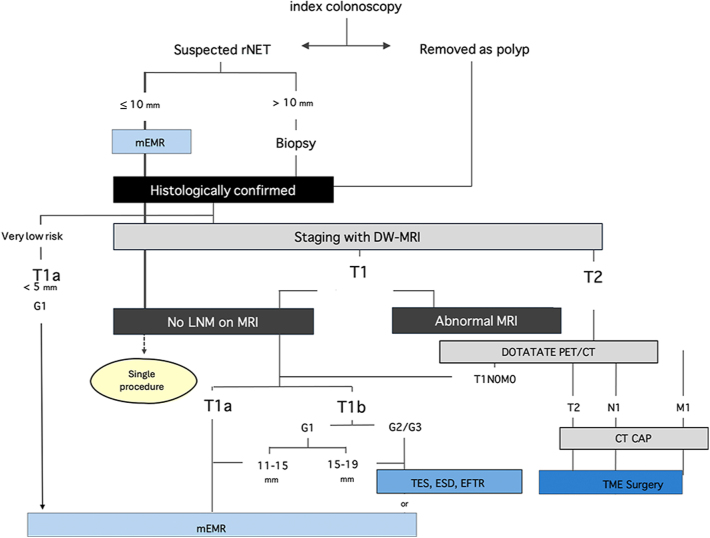
Flowchart of rectal NET staging, treatment, follow-up, and risk categories. Flowchart and summary of staging, risk stratification by grade corresponding to treatment recommendations based upon size, and surveillance recommendations based upon risk category. Color coding: gray – imaging, red – treatment, and yellow – areas of needed research. Abbreviations: AJCC, American Joint Committee on Cancer; DW-MRI, diffusion-weighted magnetic resonance imaging; G1, grade 1; G2, grade 2; G3, grade 3; M0, no metastasis; M1, presence of metastasis; mm, millimeter; N, node; N0, absence of nodal involvement; N1, lymph node metastasis; NETs, neuroendocrine tumors; yr., year, TES, transanal endoscopic surgery; ESD, endoscopic submucosal dissection; and EFTR, endoscopic full-thickness resection.

The below NANETS Guideline recommendations define the diagnosis, staging, management, and surveillance of patients with stage I–III rNETs and are based on the conclusions of the systematic literature review using GRADE methodology and consensus statements of the Delphi surveys. Diagnosis, treatment, and management recommendations are summarized in Fig. 3.

### Diagnosis and staging recommendations

The vast majority of rNETs are diagnosed with T1 presentation. The risk of nodal involvement and distant metastasis is small; however, in prospective studies, it has been shown that the risk can be up to 25% and, therefore, should be ruled out with baseline MRI staging.

– The size, depth of invasion, lymphovascular invasion, grade based on Ki-67/mitotic count index, and final tumor margin status should be specified *(grade: HIGH)*.
– L-cell immunophenotype may be determined in the pathologist report, using serotonin negativity and CDX2 positivity immunohistochemistry as surrogate markers *(grade: MODERATE)*.
– T1 tumors >5 mm should be staged with pelvic MRI *(grade: LOW)*.
– T2 tumors should be staged with pelvic MRI *(grade: HIGH)*.
– DOTATATE PET/CT should be done to define abnormalities seen on pelvic MRI and CT *(grade: HIGH)*.
– Staging for T2 tumor should include CT CAP *(grade: HIGH)*.

### Management recommendations

Current guidelines increasingly align with this evidence, recommending a more selective approach to resection. For cN0 tumors, mEMR remains the standard, while ESD, EFTR, and OTSC or transanal endoscopic surgery (TEMS and TAMIS) are considered for larger T1b tumors to ensure optimal management. Early recognition of an incomplete diagnostic resection allows for completion excision with a more advanced endoscopic technique prior to scar healing. The evolving understanding of rNET treatment underscores the necessity of balancing oncologic efficacy with procedural safety and patient-centered outcomes, favoring minimally invasive endoscopic resection and surgery over radical surgical interventions whenever feasible.

### T1

– T1 < 5 mm, grade 1 tumors can be sufficiently treated with EMR/mEMR or excisional biopsy (2–3 mm) *(grade: HIGH)*.
– T1a/b tumors can be treated with mEMR *(grade: HIGH)*.
– Larger T1b tumors can be considered for ESD, EFTR, and OTSC or transanal endoscopic surgery (TEMS and TAMIS) *(grade: MODERATE)*.
– Patients may be offered more advanced endoscopic resection or full-thickness excision surgery to achieve negative tumor margins *(grade: MODERATE)*.

### T2 +

– TME remains the standard management of T2 + tumors, regardless of clinical N stage *(grade: HIGH)*.
– Organ preservation with tumor excision to avoid radical surgery remains experimental for patients with cT2N0 tumors.
– Peri-operative therapy of locally advanced tumors with chemotherapy/radiation therapy, systemic therapy, or peptide receptor radionuclide therapy (PRRT) is not established *(grade: HIGH)*.
– Patients with unresectable, non-metastatic rNETs may be offered systemic therapy with agents approved for the treatment of advanced disease *(grade: HIGH)*.

### Surveillance recommendations

**Figure 4:**
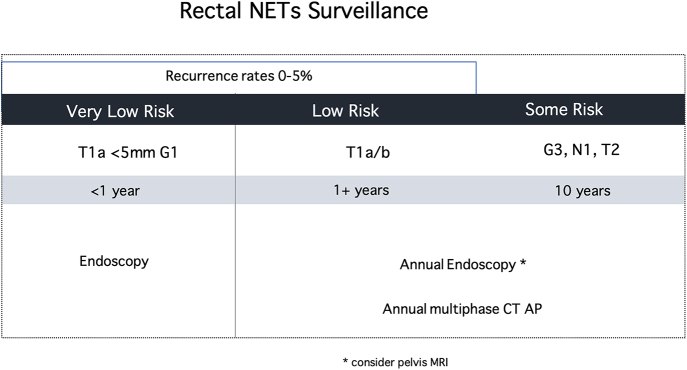
Rectal NET surveillance. Recommendations for surveillance of stage I–III rectal NETs. Abbreviations: CT AP, computed tomography of the abdomen and pelvis; G1, grade 1; G2, grade 2; G3, grade 3; mm, millimeter; N1, lymph node metastasis; NETs, neuroendocrine tumors; and MRI: magnetic resonance imaging.

Recommendations for surveillance of stage I–III rectal NETs are summarized in Fig. 4.

– T1 <5 mm G1 should be offered follow-up endoscopy within one year if treated by excisional biopsy alone *(grade: MODERATE)*.
– T1 tumors with high-risk features treated with endoscopic excision require annual rectal endoscopic surveillance and cross-sectional abdominal/pelvic imaging *(grade: LOW)*.
– Minimal duration of follow-up for patients with node-positive rNETs in multiphase CT imaging is 10 years *(grade: MODERATE)*.
– DOTATATE PET/CT should be performed if abnormalities are seen on cross-sectional imaging *(grade: HIGH)*.

## Conclusion

This guideline, developed using the GRADE methodology, updates the management of rectal NETs based on recent outcome studies and incorporates insights from long-term follow-up studies on recurrence and progression. The guideline weighs the quality of evidence alongside emerging research. A cross-sectional approach with broad search terms allowed us to identify key themes relevant to clinical practice.

The guideline refines rectal NET management to align with the latest evidence-based practices. As more real-world and prospective studies emerge, they should replace practices shaped by retrospective analyses influenced by historical treatment paradigms. The goal is to move toward more personalized, minimally invasive treatment with lower risks of morbidity and mortality. The recommendations are limited by the existing literature, particularly for tumors larger than 20 mm, where surgical intervention remains the standard. Further research is needed to determine if less invasive approaches could be beneficial for this group.

Management of rectal NETs is becoming more tailored and less invasive, improving patient outcomes. This progress reflects the collective work of researchers worldwide, whose contributions are enhancing the treatment of this rare disease.

## Supplementary materials



## Declaration of interest

The authors declare no conflict of interest that could be perceived as prejudicing the impartiality of this work.

## Funding

This work did not receive any specific grant from any funding agency in the public, commercial, or not-for-profit sector.
