# Supplementary material for: NANETS Guidelines for the diagnosis and management of stage I–III rectal neuroendocrine tumors
Source: Endocr Relat Cancer. 2026 Feb 26;33(2):e250303. doi: 10.1530/ERC-25-0303 (PMC13138514; doi:10.1530/ERC-25-0303)
Supplement: Supplementary file 1 [file supplementary_materials.pdf]

Supplementary Table 1. Methods of Resection of Rectal NETs

Legend: A comprehensive list and summary of the resection methods utilized in rNETs treatment.

Resection method	Procedure overview
**DIAGNOSTIC BIOPSY**<br>**Snare polypectomy**	A procedure that removes a polyp from the colon using a wire snare to scrape it away. The polyp is removed at its base, or stalk, without cutting into the surrounding tissue.
**ENDOSCOPIC MUCOSAL RESECTION (EMR)**	An endoscopic technique based on a submucosal injection of a saline solution to elevate the mucosal lesion away from the muscularis propria, followed by a snare cautery resection.
**Underwater endoscopic mucosal resection (UEMR)**	Performed under water immersion with the tip of a rotatable snare anchored to the proximal side of the NET to keep the NET at the center of the snare. Water was aspirated to capture as much tissue around and under the NET as possible. NETs were then cut with pure-cut mode diathermy. The mucosal defect was closed using are openable endoclip using underwater immersion, more endoclips are added if needed.
**EMR with circumferential precutting (EMR-P)**<br>*or EMR with circum. Incision (EMR-CI or CI-EMR)*	Is performed by lifting the mucosa with a saline injection, making a circumferential incision (pre-cutting) using the tip of the snare or special endoknives and resecting the tumor with a snare. This technique has no size limitation with respect to the tumor resection.
**Cap-assisted EMR (EMR-C)**	It utilizes a specialized cap with a snare to capture and remove tissues, essentially relying on the cap's design for tissue manipulation rather than direct suctioning. May or may not use suction depending on the cap design.
**EMR with a ligation device (EMR-L)**	It is performed after an initial submucosal saline injection to elevate it from the muscle layer, by suctioning the lesion into the ligating device and cutting around the concerned area with the ligation by using a round snare. A disadvantage of EMR-L may be the fact that it is only applicable for tumors of 10 mm or less in size, due to the short diameter of the caps fitted to colonoscopies.
**EMR w/ ligation & ultrasound (EMR-LUS)**	Same as EMR-L but the region surrounding the ligation band is examined by EUS to determine whether the lesion is completely ligated.
**EMR-double band (EMR-db)**	The lesion is suctioned into the ligating device, the first band is deployed to ligate the lesion and increase luminal protuberance; then the second band is deployed below the first one after endoscopic suctioning of the tumor into the cap. The lesion resection is performed via electrocautery below the second band.
**EMR- Multiple ligation w/ ultrasound (EMR-MLUS)**	Same as EMR-db but ligation is confirmed by EUS.
**Tip-in (anchor snare tip) EMR (as-EMR)**	Technique where the snare tip is deliberately anchored into the submucosa of the lesion by making a small incision, essentially securing it in place before fully opening the snare to capture and remove the tissue.
**Clip and snare assisted endoscopic submucosal resection (Cs-ESMR)**	Technique that uses endoscopic clips to elevate and stabilize a flat or difficult-to-grasp lesion, allowing for easier capture and resection with a snare during the procedure; essentially, the clips help "anchor" the tissue before using the snare to remove it.
**ENDOSCOPIC SUBMUCOSAL DISSECTION (ESD)**	An endoscope is inserted through the anus, a solution is injected under the tumor to lift it from the wall, an endoknife is used to dissect the tumor in small pieces and the tumor is removed in one piece.

**ESD w/myectomy**	Is when circumferential mucosal incisions are made using a dual knife after the submucosal injection, and submucosal with combined resection of the circular muscle layer dissection is then carried out using the dual knife or IT knife until the tumor is completely removed, and the longitudinal muscle layer was conserved. The other steps are similar to the ESD procedure.
**Pocket creation method with Hook knife (ESD-PCM)**	Utilizes a specialized electrosurgical knife for ESD that can cut the submucosa just above the muscular layer to prevent exposure of the tumor by hooking and pulling backwards when cutting the submucosal fiber, The direction of the top of the knife can be controlled and kept parallel to the muscular layer to prevent perforation.
**Rubber band traction assisted ESD**	A rubber band is used to apply traction to a lesion during the procedure, allowing for better visualization and easier dissection of the submucosal layer by attaching the band to the lesion with clips and anchoring it to the opposite wall of the gastrointestinal tract, essentially pulling the lesion taut for precise removal;
**Hybrid ESD (+ snaring)**	Hybrid ESD is a simplified technique where, after the circumferential incision around the lesion, the submucosal injection of a saline solution, and a partial dissection of the submucosa, snaring is performed using a polypectomy snare to dissect the lesion completely, instead of an endoknife.
**Dual-channel endoscope (EMR-D)**	Lifts the lesion with grasping forceps to identify the lower margin of the submucosal lesion and strangling the base with a snare. Enables deeper resection compared with standard EMR.
	**FULL THICKNESS EXCISION STRATEGIES**
**Transanal local excision (TALE)**	TALE is a surgical technique performed in the lower rectum, less than 7 cm from the anal verge and less than 1/3 lumen diameter in size; it is not used in the higher rectum as exposure is significantly limited. Introduces anal retractors into the anal canal to maintain exposure, lifts the mucosa with a saline injection to elevate the lesion and resects the tumor with electrocautery under direct vision; the defect in the rectal wall is closed with an absorbable suture. Must be performed with the patient under local or general anesthesia.
**Transanal endoscopic microsurgery (TEM)**	TEM is performed by using a multi-channel port positioned transanally that allows, at the same time, the use of a rigid rectoscope with magnified three-dimensional vision and endosurgical instruments: the scheduled resection area is previously marked by electrocautery dots, then a full-thickness resection down to the perirectal fat is performed, and finally the defect in the rectal wall is closed by a continuous running suture with clips or absorbable monofilament. As with TALE, TEM must be performed with the patient under local or general anesthesia. Has advantages over conventional transanal resection, because it provides improved operative visualization and access to lesions higher in the rectum.
**Transanal minimally invasive surgery (TAMIS)**	TAMIS utilizes a disposable port and standard laparoscopic instruments, whereas TEM uses a rigid, reusable scope, making TAMIS considered more flexible and potentially less invasive compared to TEM
**Endoscopic full thickness resection (eFTR)**	Newer technique using a full thickness resection device (FTRD). Enables deep resection to the serosal layer to be performed and then an over the scope clip ensures no perforation occurs. Procedure duration: 18.5 minutes. Could be useful when ESD in not available.
**Over The Scope Clip (OTSC), Ovesco**	Over The Scope Clip (OTSC) clip permits full thickness excision both above and below the peritoneal reflection. In contrast to common endoscopic clips, the OTSC is able to capture a larger volume of tissue with a higher compression force; thus it can be used for closure of luminal perforations larger than 10 mm.

Abbreviations: cm, centimeter; EUS, endoscopic ultrasound; FTRD, full thickness resection device; mm, millimeter; NETs, neuroendocrine tumors
